# Supplementary material for: Behavioral and cultural determinants of symptomatic dry eye disease among university students in the UAE
Source: PLoS One. 2026 Feb 26;21(2):e0343508. doi: 10.1371/journal.pone.0343508 (PMC12944768; doi:10.1371/journal.pone.0343508)
Supplement: Attachment — Submitted filename: PONE-D-25-45343.pdf [file pone.0343508.s002.pdf]

PLOS ONE

Behavioral and Cultural Determinants of Symptomatic Dry Eye Disease Among University Students in the UAE

--Manuscript Draft--

**Manuscript Number:**	PONE-D-25-45343
**Article Type:**	Research Article
**Full Title:**	Behavioral and Cultural Determinants of Symptomatic Dry Eye Disease Among University Students in the UAE
**Short Title:**	Symptomatic Dry Eye Disease Among University Students in the UAE
**Corresponding Author:**	Wissam Ghach, Ph.D.<br>Canadian University Dubai<br>Dubai, UNITED ARAB EMIRATES
**Keywords:**	Dry Eye Disease; smoking; Cosmetic use; University Students; United Arab Emirates
**Abstract:**	Objectives<br><br>This study aims to estimate the prevalence of symptomatic Dry Eye Disease (DED) and its associated risk factors among university students in the UAE.<br><br>Methods<br><br>A cross-sectional study was conducted among 654 participants using the validated Ocular Surface Disease Index (OSDI) questionnaire, distributed via an online Google survey. The survey assessed the prevalence of DED along with demographic and behavioral-cultural risk factors. Kruskal-Wallis test was employed to analyze group differences. Spearman’s rank correlation evaluated associations between OSDI scores and quantitative variables.<br><br>Results<br><br>The study revealed significant gender differences, with females reporting higher OSDI scores than males. Smoking behaviors were strongly associated with OSDI scores, with daily smokers and those smoking in indoor environments. Among smoking types, Dokha and Mouassal waterpipe users exhibited the highest median OSDI scores. Eye cosmetic practices with users of shorter annual usage reporting higher scores, while effective cleansing methods, such as soap and water or cleansing creams, were associated with lower scores. Prolonged screen time and extended study hours were positively correlated with OSDI scores.<br><br>Conclusions<br><br>Symptomatic DED is highly prevalent among UAE university students. These findings underscore the need for targeted awareness and prevention strategies to mitigate DED risk factors within this population.
**Order of Authors:**	Mona Aridi, Ph.D.
	Nisreen Alwan, Ph.D.
	Wissam Ghach, Ph.D.
**Additional Information:**	
**Question**	**Response**
**Financial Disclosure**<br><br>Enter a financial disclosure statement that describes the sources of funding for the	The author(s) received no specific funding for this work.

Powered by Editorial Manager® and ProduXion Manager® from Aries Systems Corporation

work included in this submission. Review the submission guidelines for detailed requirements. View published research articles from *PLOS ONE* for specific examples.<br><br>This statement is required for submission and **will appear in the published article** if the submission is accepted. Please make sure it is accurate.<br><br>**Funded studies**<br>Enter a statement with the following details:<br>• Initials of the authors who received each award<br>• Grant numbers awarded to each author<br>• The full name of each funder<br>• URL of each funder website<br>• Did the sponsors or funders play any role in the study design, data collection and analysis, decision to publish, or preparation of the manuscript?<br><br>Did you receive funding for this work?	
**Competing Interests**<br><br>Use the instructions below to enter a competing interest statement for this submission. On behalf of all authors, disclose any competing interests that could be perceived to bias this work—acknowledging all financial support and any other relevant financial or non-financial competing interests.<br><br>This statement is required for submission and **will appear in the published article** if the submission is accepted. Please make sure it is accurate and that any funding sources listed in your Funding Information later in the submission form are also declared in your Financial Disclosure statement.<br><br>View published research articles from *PLOS ONE* for specific examples.	The authors have declared that no competing interests exist.

**NO authors have competing interests**<br><br>Enter: *The authors have declared that no competing interests exist.*<br><br>**Authors with competing interests**<br><br>Enter competing interest details beginning with this statement:<br><br>*I have read the journal's policy and the authors of this manuscript have the following competing interests: [insert competing interests here]*<br><br>* typeset	
**Ethics Statement**<br><br>Enter an ethics statement for this submission. This statement is required if the study involved:<br><br>• Human participants<br>• Human specimens or tissue<br>• Vertebrate animals or cephalopods<br>• Vertebrate embryos or tissues<br>• Field research<br><br>Write "N/A" if the submission does not require an ethics statement.<br><br>General guidance is provided below. Consult the submission guidelines for detailed instructions. **Make sure that all information entered here is included in the Methods section of the manuscript.**	Ethical approval (2023/95) was obtained from the Institutional Review Board (IRB) at Jordan University of Science and Technology (JUST). All participants provided written informed consent to be acknowledged about the benefits, potential risks, and the confidentiality prior to their participation in the study.

Powered by Editorial Manager® and ProduXion Manager® from Aries Systems Corporation

**Format for specific study types**<br><br>**Human Subject Research (involving human participants and/or tissue)**<br>• Give the name of the institutional review board or ethics committee that approved the study<br>• Include the approval number and/or a statement indicating approval of this research<br>• Indicate the form of consent obtained (written/oral) or the reason that consent was not obtained (e.g. the data were analyzed anonymously)<br><br>**Animal Research (involving vertebrate animals, embryos or tissues)**<br>• Provide the name of the Institutional Animal Care and Use Committee (IACUC) or other relevant ethics board that reviewed the study protocol, and indicate whether they approved this research or granted a formal waiver of ethical approval<br>• Include an approval number if one was obtained<br>• If the study involved *non-human primates*, add *additional details* about animal welfare and steps taken to ameliorate suffering<br>• If anesthesia, euthanasia, or any kind of animal sacrifice is part of the study, include briefly which substances and/or methods were applied<br><br>**Field Research**<br><br>Include the following details if this study involves the collection of plant, animal, or other materials from a natural setting:<br>• Field permit number<br>• Name of the institution or relevant body that granted permission	
**Data Availability**<br><br>Authors are required to make all data underlying the findings described fully available, without restriction, and from the time of publication. PLOS allows rare exceptions to address legal and ethical concerns. See the PLOS Data Policy and FAQ for detailed information.	Yes - all data are fully available without restriction

A Data Availability Statement describing where the data can be found is required at submission. Your answers to this question constitute the Data Availability Statement and **will be published in the article**, if accepted.<br><br>**Important:** Stating 'data available on request from the author' is not sufficient. If your data are only available upon request, select 'No' for the first question and explain your exceptional situation in the text box.<br><br>Do the authors confirm that all data underlying the findings described in their manuscript are fully available without restriction?	
**Describe where the data may be found in full sentences. If you are copying our sample text, replace any instances of XXX with the appropriate details.**<br><br>• If the data are **held or will be held in a public repository**, include URLs, accession numbers or DOIs. If this information will only be available after acceptance, indicate this by ticking the box below. For example: *All XXX files are available from the XXX database (accession number(s) XXX, XXX.).*<br>• If the data are all contained **within the manuscript and/or Supporting Information files**, enter the following: *All relevant data are within the manuscript and its Supporting Information files.*<br>• If neither of these applies but you are able to provide **details of access elsewhere**, with or without limitations, please do so. For example:<br><br>*Data cannot be shared publicly because of [XXX]. Data are available from the XXX Institutional Data Access / Ethics Committee (contact via XXX) for researchers who meet the criteria for access to confidential data.*<br><br>*The data underlying the results presented in the study are available from (include the name of the third party*	All relevant data are within the manuscript and its Supporting Information files.

and contact information or URL).<br>• This text is appropriate if the data are owned by a third party and authors do not have permission to share the data.<br><br>* typeset	
Additional data availability information:	Tick here if the URLs/accession numbers/DOIs will be available only after acceptance of the manuscript for publication so that we can ensure their inclusion before publication.

Powered by Editorial Manager® and ProduXion Manager® from Aries Systems Corporation

Manuscript
Click here to access/download;Manuscript;Manuscript_UAE.docx

Behavioral and Cultural Determinants of Symptomatic Dry Eye Disease Among University Students in the UAE

Mona Aridi Ph.D. [1], Nisreen Alwan Ph.D. [2,3], Wissam Ghach, Ph.D.[4*]

[1] Univ Angers, LARIS, SFR MATHSTIC, F-49000 Angers, 62 avenue Notre Dame du Lac, 49000 Angers, France.
[2] College of Health Sciences, Abu Dhabi University, Abu Dhabi, United Arab Emirates.
[3] Vision Care Association, Beirut, Lebanon.
[4] Department of Public Health, Canadian University Dubai, Dubai, United Arab Emirates

Corresponding author:
Wissam Ghach, Department of Public Health, Canadian University Dubai, UAE
Email: wissam.ghach@cud.ac.ae

Abstract

Objectives : This study aims to estimate the prevalence of symptomatic Dry Eye Disease (DED) and its associated risk factors among university students in the UAE. **Methods:** A cross-sectional study was conducted among 654 participants using the validated Ocular Surface Disease Index (OSDI) questionnaire, distributed via an online Google survey. The survey assessed the prevalence of DED along with demographic and behavioral-cultural risk factors. Kruskal-Wallis test was employed to analyze group differences. Spearman's rank correlation evaluated associations between OSDI scores and quantitative variables. **Results:** The study revealed significant gender differences, with females reporting higher OSDI scores than males. Smoking behaviors were strongly associated with OSDI scores, with daily smokers and those smoking in indoor environments. Among smoking types, Dokha and Mouassal waterpipe users exhibited the highest median OSDI scores. Eye cosmetic practices with users of shorter annual usage reporting higher scores, while effective cleansing methods, such as soap and water or cleansing creams, were associated with lower scores. Prolonged screen time and extended study hours were positively correlated with OSDI scores. **Conclusions:** Symptomatic DED is highly prevalent among UAE university students. These findings underscore the need for targeted awareness and prevention strategies to mitigate DED risk factors within this population.

Keywords: *Dry Eye Disease; Smoking; Cosmetic use; University Students; United Arab Emirates*

1. Introduction

Dry Eye Disease (DED) is a multifactorial condition of the ocular surface that disrupts tear film homeostasis and is accompanied by ocular symptoms. The Tear Film and Ocular Surface Society Dry Eye Workshop II (TFOS DEWS II) defines DED as "a loss of homeostasis of the tear film accompanied by tear film instability, hyperosmolarity, ocular surface inflammation, damage, and neurosensory abnormalities" [1]. The prevalence of DED is significant, ranging globally from 5% to 50%, with Arab countries reporting some of the highest rates [2] particularly, among the Jordanian population [3]. Likewise, the prevalence of DED in Middle Eastern countries is higher than the global estimate, highlighting a significant regional variation [4]. This variability is attributed to factors such as geography, climate, and diagnostic criteria, highlighting the importance of region-specific investigations.

DED prevalence is typically assessed using clinical tools such as Tear Break-Up Time (TBUT) and Schirmer Test, or symptom-based questionnaires, including the Ocular Surface Disease Index (OSDI) and Standard Patient Evaluation of Eye Dryness (SPEED) [5,6]. In addition to environmental and genetic factors, behavioral and cultural habits, including smoking and eye cosmetic use, have emerged as critical risk factors [3,7,8].

Smoking remains a significant global public health issue, with tobacco products containing over 4,000 toxic chemicals that contribute to cancers, cardiovascular diseases, and ocular conditions [9]. Smoking has been shown to destabilize tear film, impair ocular surface defenses, and disrupt the retinal nerve fiber layer [10,11]. Similarly, the widespread use of eye cosmetics poses potential risks to ocular health. Ingredients in cosmetics, particularly mascara and internal eyeliner, may alter the lipid layer of the tear film, leading to discomfort and exacerbating DED [12]. While tobacco and cosmetics are predominantly used, limited research has explored their contribution

to DED prevalence in the UAE, leaving a critical gap in understanding gender-specific risk factors.

This study seeks to address these gaps by estimating the prevalence of symptomatic DED among university students in the UAE and investigating its association with behavioral and cultural risk factors, such as smoking and cosmetic use, using the validated OSDI questionnaire. By providing region-specific data, this research aims to raise awareness among students and inform the development of community-based interventions and awareness campaigns. The findings are expected to guide local authorities, non-governmental organizations, and eye health professionals in designing educational programs to mitigate DED risk factors.

This study is the first to comprehensively examine the prevalence of symptomatic DED and its association with behavioral and cultural risk factors among university students in the United Arab Emirates (UAE). It uniquely investigates the interplay between DED and lifestyle practices, such as smoking behaviors (*e.g.* Dokha, Ajami), the duration of daily electronic device use, and the number of studying hours per day. Furthermore, the study explores the effect of eye cosmetics use, including long-term application and hygiene practices on DED severity. By focusing on young university students—a demographic increasingly exposed to digital screens, academic stress, and cultural practices, this research fills a critical gap in understanding how these factors influence DED prevalence and severity. Utilizing the validated OSDI and robust statistical analyses, the study provides novel insights that are expected to inform targeted awareness campaigns and intervention strategies tailored to this population.

2. Methods

2.1 Study Design and Population

A cross-sectional study was conducted between April 2023 and December 2023 to assess DED prevalence among university students in the UAE. Participants were recruited through a Co[illegible]ience Sampling Technique, with a Google survey distributed via social media platforms and research departments in UAE universities. The study population comprised 654 university students, stratified by variables such as gender, age, residence, field of study, study level, daily study duration, electronic device usage, contact lens wear, use of moisturizing eye drops, and current diagnosis of eye dryness. Exclusion criteria included individuals under 18 years old, non-university students, and those with eye surgeries, active ocular diseases, or medications affecting tear film production or ocular surface integrity.

2.2 Study Tool: Ocular Surface Disease Index (OSDI)

The OSDI questionnaire, developed by the Outcomes Research Group at Allergan Inc., is a validated instrument designed to assess symptoms related to eye irritation and DED [5]. It consists of 12 items divided into three sections: ocular symptoms, vision-related function, and environmental triggers. Each item is graded on a scale from 0 (none of the time) to 4 (all of the time). The total OSDI score is calculated using the formula:

$$OSDI\ Score = \frac{Sum\ of\ scores\ for\ all\ questions\ answered\ \times 100}{Total\ number\ of\ questions\ answered\ \times 4}$$

Scores are classified into three categories: normal [0–12], mild-to-moderate [13–32], and severe [33–100].

2.3 Assessment of Behavioral and Cultural Risk Factors

The study evaluates various behavioral and cultural risk factors associated with the prevalence and severity of DED among the study population. Smoking behaviors assessed include the types of tobacco used—such as regular cigarettes, electronic cigarettes, Medwakh pipes, Mouassal waterpipes (flavored and honeyed tobacco/glycerin paste), and Tumbak/Ajami waterpipes (non-honeyed pure dark tobacco paste)—as well as smoking frequency (daily, weekly, or monthly) and smoking environment (indoors or outdoors). Additionally, smoking quantifi[illegible]n and duration are examined by considering the number of cigarettes, waterpipes, or Medwakh pipes smoked per week and the total years of smoking. Eye cosmetic use is also analyzed, focusing on the types of products (mascara, eyeliner, eyeshadow, eyelashes), duration of use (<6 months, 6–12 months, >12 months), daily application time (in hours), cleaning methods (water, soapy water, or cleansing products), frequency of night cleansing (never, sometimes, always), and sleep routines (with or without lenses and/or eye cosmetics). These variables are investigated to identify their relationship with the development and severity of DED.

2.4 Data Analysis

Data analysis was performed using the Statistical Package for the Social Sciences (SPSS), version 21 (IBM Corp., Chicago, IL, USA). The prevalence of DED was expressed in % of participants with an OSDI score ≥ 13, encompassing mild, moderate, and severe OSDI categories.

The OSDI questionnaire assessed the severity of DED symptoms, with higher scores indicating more severe symptoms. Behavioral variables included the number of smoking years, frequency

and type of eye cosmetics use, and daily habits such as the number of study hours and time spent using electronic devices.

The normality of the data was assessed using the Shapiro-Wilk & Kolmogorov-Smirnov tests, and all tested variables were found to be non-normally distributed ($P < 0.05$). Descriptive statistics were calculated using the median and Interquartile Range (IQR) to summarize the non-normally distributed continuous variables. To analyze differences in OSDI scores based on categorical variables (*e.g.* smoking status and frequency of eye cosmetics use), the Kruskal-Wallis test was applied, with results reported as the median, IQR, H-value, and P-value. The association between OSDI scores and quantitative variables such as the number of smoking years, study hours, and electronic device usage was assessed using Spearman's rank correlation coefficient, with correlation coefficients (ρ) and P-values reported. A significance level of 0.05 ($P < 0.05$) was adopted for all statistical tests, with 95% confidence intervals reported where applicable.

2.5 Ethical Considerations

Ethical approval (2023/95) was obtained from the Institutional Review Board (IRB) at Jordan University of Science and Technology (JUST). All participants provided written informed consent to be acknowledged about the benefits, potential risks, and the confidentiality prior to their participation in the study.

3. Results

3.1 DED and Participants' Profiles

A total of 654 university students participated in this study. The majority were females (54.1%) and aged between 18 and 21 years (48.6%). Regarding the participants' field and year of study,

the highest participation was from undergraduate levels (92.7%) mainly from engineering students (24.6%) and business and tourism students (29.1%). Most participants were non-smokers (58.9%) and did not wear contact lenses (66.2%). Although 82% of the participants had not been clinically diagnosed with DED. According to the symptomatic assessment of DED by OSDI, 47.1% of the participants (47[illegible]) reported mild-to-severe DED (Table 1).

The [illegible]ality of the data was assessed using the Shapiro-Wilk and Kolmogorov-Sminrnov tests, which indicated that the distribution of all variables under study was not normally distributed ($P < 0.05$). Consequently, the Kruskal-Wallis test was employed instead of ANOVA to examine the if significant differences existed in OSDI scores among variables. The results are presented in Table 1, as median, IQR, H-values, and corresponding significance levels (P-values).

The Kruskal-Wallis test revealed significant differences in OSDI scores based on gender ($H = 12.569$, $P < 0.001$), with females reporting higher median OSDI scores (14.58, IQR: 22.92) than males (10.42, IQR: 16.66). However, no significant differences were observed across age intervals ($H = 2.026$, $P = 0.567$), with median OSDI scores ranging from 12.50 (IQR: 18.75) in the 18–23 age groups to 14.58 (IQR: 22.95) in those aged 24–26, and 13.54 (IQR: 31.77) in participants aged ≥ 27.

[illegible] field of study showed no significant differences in OSDI scores ($H = 13.622$, $P = 0.058$), although participants from Art and Media Sciences reported the highest median score (16.67, IQR: 22.92), while those in Humanities and Political Sciences had the lowest (5.21, IQR: 9.37). Similarly, the level of study did not exhibit significant differences ($H = 6.649$, $p = 0.355$), with median OSDI scores ranging from 11.45 (IQR: 16.67) in first-year bachelor’s students to 14.58 (IQR: 27.08) in fourth-year bachelor’s students.

A significant difference existed in OSDI scores among smoking habits (H = 26.721, P < 0.001), as smokers had higher median scores (16.67, IQR: 25.00) compared to non-smokers (10.42, IQR: 18.75). Contact lens use was also significantly associated (H = 39.986, P < 0.001), with those always using lenses reporting the highest median OSDI score (22.92, IQR: 27.08), compared to those who sometimes (18.75, IQR: 22.92) or never (10.42, IQR: 16.66) used lenses.

Finally, eye dryness was significantly related to OSDI scores (H = 56.664, P < 0.001). Participants reporting eye dryness had markedly higher median scores (25.00, IQR: 31.25) than those without dryness (10.42, IQR: 18.75).

3.2 Smoking Profile

The Kruskal-Wallis test was conducted to assess if there is a significant different in OSDI scores among smoking variables, as the Shapiro-Wilk/Kolmogorov-Smirnov tests indicated a non-normal distribution for all variables (P < 0.05). The results are presented in Table 2 as median (IQR), H-values, and significance levels (P-values).

There was a significant different in [illegible] scores among smoking types. (H = 31.688, P < 0.001). Non-smokers reported the lowest median OSDI score (4.17, IQR: 10.42), while smokers using dokha (20.83, IQR: 31.25) and waterpipe (mouassal) (20.83, IQR: 39.59) had the highest scores. Regular cigarette smokers had a median score of 16.67 (IQR: 35.41), electronic cigarette (vape) users had 14.58 (IQR: 18.75), and waterpipe (ajami) users reported 10.42 (IQR: 29.16).

The smoking rate also significantly influenced OSDI scores (H = 25.737, P < 0.001). Participants who smoked daily had the highest median OSDI score (17.71, IQR: 29.69), followed by weekly smokers (16.67, IQR: 21.36) and monthly smokers (12.50, IQR: 14.59), with non-smokers reporting a median score of 10.42 (IQR: 18.75).

Smoking areas were significantly associated with OSDI scores (H = 34.491, P < 0.001). Participants who smoked indoors reported the highest median OSDI score (27.08, IQR: 31.76), followed by those who smoked in mixed areas (16.17, IQR: 22.40) and outdoor smokers (12.50, IQR: 20.83). Non-smokers again had the lowest scores, with a median of 10.42 (IQR: 18.75).

3.2 The Usage of the Eye Cosmetic Products

The Kruskal-Wallis test was performed to evaluate if significant differences exist in OSDI scores among eye cosmetics usages, as the normality test indicated a non-normal distribution for all variables (P < 0.05). Results were expressed as median (IQR), H-values, and significance levels (P-values) in Table 3.

Inner eyeliner usage frequency significantly influenced OSDI scores (H = 17.329, P = 0.002). Participants who applied inner eyeliner daily had the highest median OSDI score (19.79, IQR: 27.09), followed by those using it 1–2 time(s) per week (16.67, IQR: 28.64), 1–2 time(s) per month (16.67, IQR: 21.87), or 3–4 times per week (14.58, IQR: 15.62). Those wh[illegible]er used inner eyeliner had the lowest median score (12.50, IQR: 18.75).

External eyeliner usage also showed a significant effect on OSDI scores (H = 23.263, P < 0.001). Daily users had a median score of 16.67 (IQR: 26.04), similar to those using it 3–4 times per week (16.67, IQR: 28.12) or 1–2 time(s) per week (15.63, IQR: 29.17). Occasional users (1–2 time(s) per month) reported a median of 12.50 (IQR: 20.83), while those who never used external eyeliner had the lowest scores (10.42, IQR: 16.66).

False eyelash usage was significantly associated with OSDI scores (H = 22.444, P < 0.001). Participants using false eyelashes 3–4 times per week had the highest median score (33.33, IQR: 40.63), followed by those using them 1–2 time(s) per month (21.87, IQR: 28.13) or 1–2

time(s) per week (20.83, IQR: 29.17). Daily users reported a median of 14.58 (IQR: 48.96), and non-users had the lowest scores (12.50, IQR: 16.67).

Eye s[illegible]w usage was another significant factor (H = 17.001, P = 0.002). Daily users had the highest median OSDI score (17.71, IQR: 24.48), followed by 1–2 time(s) per week users (16.67, IQR: 26.04), and 1–2 time(s) per month users (14.58, IQR: 20.32). Participants using eye shadow 3–4 times per week had a median score of 12.50 (IQR: 22.92), while non-users reported the lowest scores (10.42, IQR: 17.18).

Significant differences also existed in OSDI scores among Mascara usage (H = 20.234, p < 0.001). Participants using mascara 1–2 time(s) per month had the highest median score (18.75, IQR: 37.50), followed by 1–2 time(s) per week users (16.67, IQR: 31.25), daily users (14.58, IQR: 22.92), and 3–4 times per week users (14.58, IQR: 18.75). Non-users had the lowest scores (10.42, IQR: 16.66).

3.3 Eye Cosmetics Routines and Practices

The Kruskal-Wallis test was conducted to examine the association between eye cosmetics practices and OSDI scores, as the normality tests revealed a non-normal distribution for all variables (P < 0.05). Results are presented in Table 4 as median (IQR), H-values, and significance levels (P-values).

The duration of cosmetics use per year significantly influenced OSDI scores (H = 22.628, P < 0.001). Pa[illegible]ants using cosmetics for 6-12 months reported the highest median score (18.75, IQR: 9.17), followed by those using cosmetics for less than 6 months (16.67, IQR: 25.00) or more than 12 months (14.58, IQR: 20.83). Those who did not use cosmetics or had no applicable data had the lowest scores (10.42, IQR: 16.66).

The duration of cosmetics use per day also showed a significant effect (H = 18.821, P < 0.001). Participants using cosmetics for less than 6 hours daily had a median score of 16.67 (IQR: 20.32), while those using cosmetics for 6-12 hours or more than 12 hours had scores of 14.58 (IQR: 33.33) and 14.58 (IQR: 20.83), respectively. Non-users or those without applicable data had the lowest scores (10.42, IQR: 16.66).

Cleansing techniques were significantly associated with OSDI scores (H = 22.990, P < 0.001). Participants using only water reported the highest median score (17.71, IQR: 34.90), followed by those using cleansing cream (14.58, IQR: 22.92) and soap and water (12.50, IQR: 18.75). Non-users or those without applicable data had the lowest scores (10.42, IQR: 16.66).

The rate of cleansing per day was another significant factor (H = 35.622, P < 0.001). Participants who always cleansed their cosmetics reported the highest median OSDI score (33.33, IQR: 27.08), followed by those cleansing sometimes (14.58, IQR: 31.25) or rarely (14.58, IQR: 20.83). Non-users or those with no applicable data had the lowest scores (10.42, IQR: 16.66).

Regarding sleep routines, although not statistically significant (H = 6.937, P = 0.074), participants who slept with both eye cosmetics and [illegible]es had the highest OSDI scores (52.08, IQR: 67.71), followed by those who slept only with eye cosmetics (23.96, IQR: 40.62) and those who slept with lenses (28.13, IQR: 33.85). Participants who slept without lenses and cosmetics or had no applicable data had the lowest median scores (12.50, IQR: 18.75).

The results of Spearman's rank correlation analysis indicate several significant association between the OSDI score and the different variables as presented in Table 5.

There is a strong positive correlation between the OSDI score and the number of hours spent using electronic devices per day (correlation coefficient = 0.694, P < 0.001). This suggests that as the hours spent using electronic devices increases, the OSDI score, which measures eye

dryness and discomfort, tends to increase as well. Similarly, there is a strong positive correlation between the OSDI score and the number of hours spent studying per day (correlation coefficient = 0.661, P < 0.001). This finding implies that longer study hours are associated with higher OSDI scores, possibly indicating more eye discomfort.

The analysis also reveals a moderate positive correlation between the OSDI score and the number of years a person has been smoking (correlation coefficient = 0.38, P < 0.001). This suggests that individuals who have smoked for more years tend to report higher OSDI scores, potentially indicating a link between smoking history and eye discomfort. In contrast, the correlation between the OSDI score and the number of cigarettes smoked per week is weak (correlation coefficient = 0.175, P < 0.001), but still statistically significant, suggesting that a higher number of cigarettes smoked per week is associated with a slight increase in eye discomfort.

The correlation between the OSDI score and the number of Dokha smoked per week is negligible (correlation coefficient = 0.005, P = 0.896), indicating that Dokha smoking has no significant effect on eye discomfort. Lastly, the correlation between the OSDI score and the number of Shisha smoked per week is weak (correlation coefficient = 0.097, P = 0.013). Although the correlation is weak, it is statistically significant, suggesting that higher Shisha consumption may be slightly associated with increased eye discomfort.

Overall, the strongest correlations are observed with electronic device usage and studying hours, both of which showed strong positive associations with OSDI scores. Smoking, especially the number of smoking years, also shows a moderate correlation with eye discomfort. However, Dokha smoking does not appear to affect eye discomfort, and the effect of Shisha smoking is weak but significant.

4. Discussion

The discussion of these findings can delve into several key aspects regarding the association between smoking behaviors and OSDI scores. The significant associations between smoking profiles and OSDI scores highlight the potential impact of various smoking behaviors on dry eye symptoms and severity.

4.1 Gender, Smoking Habits, and Eye Dryness

The results reveal a gender difference, with females reporting higher OSDI scores than males. This may be attributed to hormonal differences, which are known to influence tear film stability and the prevalence of DED [13,14]. Smoking habits also demonstrated a strong association with OSDI scores, where smokers had significantly higher scores than non-smokers [15]. Among smoking types, Dokha and Mouassal waterpipe users exhibited the highest median OSDI scores. This aligns with existing literature that links smoking to oxidative stress, reduced tear film quality, and inflammation, exacerbating dry eye symptoms [16,17].

4.2 Frequency and Environment of Smoking

Daily smokers reported the highest OSDI scores compared to weekly or monthly smokers, indicating a dose-response relationship between smoking frequency and the severity of ocular discomfort [18]. Moreover, smoking in indoor environments led to the highest OSDI scores. This underscores the compounded effect of poor ventilation and higher exposure to smoke particulates in closed settings, which could aggravate ocular surface damage [19].

The results emphasize the importance of smoking cessation and mitigating indoor smoking environments as preventive measures for reducing DED risk. Public health interventions could target high-risk groups, such as frequent smokers and individuals exposed to smoke indoors.

Furthermore, raising awareness about the detrimental ocular effects of smoking, including specific tobacco products like Dokha and Mouassal, could aid in behavioral changes.

4.3 Eye Cosmetics Routine and Practices

Research indicates that the use of eye cosmetics can influence ocular comfort and may be associated with dry eye symptoms. A study by Ng et al. (2012) found that while OSDI scores were similar between cosmetics users and non-users, perceived comfort was greater when cosmetics were not used [20]. Users with shorter annual usage durations (< 6 months) reported higher OSDI scores compared to those using cosmetics for over a year. This could be attributed to long-term users' adaptation or improved selection of products compatible with sensitive eyes. Non-users consistently showed the lowest OSDI scores, reinforcing the idea that avoiding cosmetics altogether reduces the risk of ocular surface discomfort.

Surprisingly, extended daily use (> 12 hours) did not significantly elevate OSDI scores compared to moderate use (6-12 hours). This might suggest that discomfort is more influenced by the quality of products and application techniques than by duration alone. Non-users reported the lowest discomfort, consistent with the protective effect of avoiding cosmetics.

The cleansing method significantly influenced OSDI scores. Participants who used water alone for removal experienced the highest discomfort, likely due to the inadequate removal of cosmetic residues, leading to prolonged irritation [12,21] Conversely, those using soap and water or cleansing creams reported lower scores, suggesting that effective cleansing minimizes residue buildup and irritation.

Frequent cleansing (*e.g.* "always") was associated with the highest OSDI scores, potentially due to overexposure to harsh chemicals or mechanical irritation from repeated rubbing. This finding

highlights the need for balanced hygiene practices that avoid excessive cleansing while ensuring proper removal of cosmetics [3].

Although not statistically significant, the trend indicates that sleeping with both cosmetics and lenses led to the highest discomfort, underscoring the importance of proper removal before rest. This behavior likely exacerbates irritation by trapping residues and reducing tear film replenishment during sleep.

4.4 Lifestyle Factors

A strong positive correlation between screen time and OSDI scores reflects the well-documented impact of digital eye strain. Prolonged exposure to screens reduces blink rates and destabilizes the tear film, leading to dryness and discomfort [22].

Similar to device usage, extended study hours were strongly correlated with higher OSDI scores. This may be due to sustained focus on reading or screens, further exacerbating dry eye symptoms.

Smoking, particularly over a longer duration, was moderately associated with higher OSDI scores. Cigarette smoke contains irritants that can destabilize the tear film and increase oxidative stress, both of which contribute to ocular surface discomfort. Shisha smoking showed a weaker correlation, possibly due to less frequent usage, while Dokha smoking had no significant impact, perhaps due to differences in exposure levels or study sample characteristics.

This study has several limitations. Data collection was based on an online self-reported questionnaire, which may introduce response bias or social desirability effects, potentially influencing the findings. Also, The estimation of DED was entirely subjective and may not align with clinical sign. Therefore, further research is recommended to clinically assess the

prevalence and severity of DED and explore its statistical association with tobacco and cosmetic use.

5. Conclusion

This study provides valuable insights into the associations between eye cosmetics practices, smoking behaviors, lifestyle factors, and ocular surface discomfort, as reflected by OSDI scores. Key findings include the role of gender, smoking frequency, and environmental factors in exacerbating symptoms of DED. Additionally, improper cleansing routines and prolonged exposure to screens further contributed to higher OSDI scores. These results underscore the multifactorial nature of ocular surface discomfort and emphasize the importance of public awareness and preventive measures.

Recommendations for future interventions include promoting smoking cessation, advocating for improved indoor air quality, and encouraging balanced hygiene practices for cosmetics users. Public health campaigns targeting high-risk groups, such as smokers and individuals with extended screen time or poor eye care habits, could help mitigate the prevalence and severity of DED.

6. Acknowledgments

The authors gratefully acknowledge Prof. May Bakkar for her technical support for the work. Acknowledgment is also given to the Canadian University Dubai (CUD) for approving and supervising the study design and protocol. Sincere gratitude is extended to all participants who contributed to this study.

7. References

1. Craig JP, Nelson JD, Azar DT, Belmonte C, Bron AJ, Chauhan SK, Paiva CS De, Hammitt KM, Jones L, Nichols JJ, Nichols KK, Novack GD, Stapleton FJ, Willcox MDP, Wolffsohn JS, Sullivan DA. The Ocular Surface TFOS DEWS II Report Executive Summary. Ocul Surf 2017;15:802–12.
2. Stapleton F, Alves M, Bunya VY, Jalbert I, Lekhanont K, Malet F, Na K-S, Schaumberg D, Uchino M, Vehof J, Viso E, Vitale S, Jones L. TFOS DEWS II Epidemiology Report. Ocul Surf 2017;15:334–65.
3. Ghach W, Bakkar MM, Aridi M, Beshtawi I, Doughaily R, Al-Fayoumi N. Prevalence and Behavioral-Based Risk Factors (Eye Cosmetic and Tobacco Use) of Symptomatic Dry Eye Disease in Four Middle Eastern Countries: Lebanon, Syria, Jordan, and Palestine. Clin Ophthalmol 2022:3851–60.
4. Craig JP, Wang MTM, Craig JP. Investigating the effect of eye cosmetics on the tear film: current insights. Clin Optom 2018;10:33.
5. Schiffman RM, Christianson MD, Jacobsen G, Hirsch JD, Reis BL. Reliability and Validity of the Ocular Surface Disease Index. Arch Ophthalmol 2000;118:615–21.
6. Özcura F, Aydin S, Helvaci MR. Ocular surface disease index for the diagnosis of dry eye syndrome. Ocul Immunol Inflamm 2007;15:389–93.
7. Al-Dossary SK. Environmental and Occupational Triggers of Dry Eye Symptoms in the Ahsa Region of Saudi Arabia: A Cross-Sectional Study. Clin Ophthalmol 2024:2427–38.
8. Britten-Jones AC, Wang MTM, Samuels I, Jennings C, Stapleton F, Craig JP. Epidemiology and risk factors of dry eye disease: considerations for clinical management. Medicina (B Aires) 2024;60:1458.
9. World Health Organization. Tobacco: Key Facts.

10. Kulkarni A, Banait S. Through the Smoke: An In-Depth Review on Cigarette Smoking and Its Impact on Ocular Health. Cureus 2023;15.

11. Thomas J, Jacob GP, Abraham L, Noushad B, Jacob GP. The effect of smoking on the ocular surface and the precorneal tear film. Australas Med J 2012;5:221–6.

12. Ng A, Evans K, North R V, Jones L, Purslow C. Impact of eye cosmetics on the eye, adnexa, and ocular surface. Eye Contact Lens Sci Clin Pract 2016;42:211–20.

13. Vasović DD, Karamarković ML, Jovanović M, Stojičić M, Kalezić T, Colić M, Musić N, Dragišić M, Jeremić M, Rašić DM. Gender-related differences in dry eye symptoms following involutional ectropion and entropion surgery. Life 2024;14:815.

14. Sherry A, Aridi M, Ghach W. Prevalence and risk factors of symptomatic dry eye disease in Lebanon. Contact Lens Anterior Eye 2020;43:355–8.

15. Bhutia P, Sen S, Nath T, Shamshad MA. The effect of smoking on ocular surface and tear film based on clinical examination and optical coherence tomography. Indian J Ophthalmol 2021;69:1693–6.

16. Altinors DD, Akça S, Akova YA, Bilezikçi B, Goto E, Dogru M, Tsubota K. Smoking associated with damage to the lipid layer of the ocular surface. Am J Ophthalmol 2006;141:1016–21.

17. Bakkar MM, Haddad MF, Khabour OF. The effects of tobacco waterpipe smoking on the ocular surface. Clin Exp Optom 2021:1–7.

18. Cheng ACK, Pang CP, Leung ATS, Chua JKH, Fan DSP, Lam DSC. The association between cigarette smoking and ocular diseases. Hong Kong Med J 2000;6:195.

19. Patel S, Mittal R, Kumar N, Galor A. The environment and dry eye—manifestations, mechanisms, and more. Front Toxicol 2023;5:1173683.

20. Ng A, Evans K, North R, Purslow C. Eye cosmetic usage and associated ocular comfort. Ophthalmic Physiol Opt 2012;32:501–7.

21. Ng A, Evans K, North R, Purslow C. The effects of cosmetic eye pencil application on the tear film and ocular surface. Invest Ophthalmol Vis Sci 2013;54:952.

22. Al-Mohtaseb Z, Schachter S, Shen Lee B, Garlich J, Trattler W. The relationship between dry eye disease and digital screen use. Clin Ophthalmol 2021:3811–20.

Tables:

Table 1: Kruskal-Wallis test results for the demographics section.

Variable		Frequency (%)	Median OSDI (IQR)	Kruskal-Wallis Test*
**Gender**	**Male**	300 (45.9%)	10.42 (16.66)	H = 12.569
	**Female**	354 (54.1%)	14.58 (22.92)	**P < 0.001**
**Age Interval**	**18-20**	318 (48.6%)	12.50 (18.75)	
	**21-23**	243 (37.2%)	12.50 (18.75)	H = 2.026
	**24-26**	63 (9.6%)	14.58 (22.95)	P = 0.567
	**≥ 27**	30 (4.6%)	13.54 (31.77)	
**Field of Study**	**Natural Sciences**	19 (2.9%)	12.50 (18.75)	
	**Health Sciences**	83 (12.7%)	12.50 (22.92)	H = 13.622
	**Medical and Pharmaceutical Sciences**	28 (4.3%)	13.54 (30.20)	P = 0.058
	**Engineering Sciences**	161 (24.6%)	10.42 (16.66)	
	**Art and Media Sciences**	127 (19.4%)	16.67 (22.92)	
	**Business and Tourism**	190 (29.1%)	12.50 (20.83)	
	**Education and Social Sciences**	38 (5.8%)	15.62 (15.63)	
	**Humanities and Political Sciences**	8 (1.2%)	5.21 (9.37)	
**Level of Study**	**Bachelor (1st year)**	184 (28.1%)	11.45 (16.67)	H = 6.649
	**Bachelor (2nd year)**	137 (20.9%)	12.50 (22.91)	P = 0.355
	**Bachelor (3rd year)**	171 (26.1%)	12.50 (18.75)	
	**Bachelor (4th year)**	115 (17.6%)	14.58 (27.08)	
	**Master (1st year) or equivalent**	43 (6.6%)	12.50 (22.91)	
	**Master (2nd year) or equivalent**	3 (0.5%)	12.50	
	**Ph.D. or equivalent**	1 (0.2%)	---	
**Smoking Habits**	**Non-smokers**	385 (58.9%)	10.42 (18.75)	H =35.881
	**Smokers**	269 (41.1%)	16.67 (25.00)	**P < 0.001**
**Contact Lens (CL) use**	**Never**	433 (66.2%)	10.42 (16.66)	H = 39.986
	**Sometimes**	157 (24.0%)	18.75 (22.92)	**P < 0.001**
	**Always**	64 (9.8%)	22.92 (27.08)	
**Eye Dryness**	**No**	536 (82.0%)	10.42 (18.75)	H = 56.664
	**Yes**	118 (18.0%)	25.00 (31.25)	**P < 0.001**
**OSDI Status**	**Normal**	346 (52.9%)		
	**Mild-to-Moderate**	193 (29.5%)		
	**Severe**	115 (17.6%)		

* Values in bold indicate significant difference ($P < 0.05$).

Table 2: Kruskal-Wallis test results for the smoking profile.

Variable		Median OSDI (IQR)	Kruskal-Wallis Test*
**Smoking Types**	**Regular Cigarette**	16.67 (35.41)	H = 31.688 **P < 0.001**
	**Electronic Cigarette (vape)**	14.58 (18.75)	
	**Dokha (medwakh pipe)**	20.83 (31.25)	
	**Waterpipe (mouassal)**	20.83 (39.59)	
	**Waterpipe (ajami)**	10.42 (29.16)	
	**Non-smoker**	4.17 (10.42)	
**Smoking Rate**	**Daily**	17.71 (29.69)	H = 25.737 **P < 0.001**
	**Weekly**	16.67 (21.36)	
	**Monthly**	12.50 (14.59)	
	**Non-smoker**	10.42 (18.75)	
**Smoking Areas**	**Indoor**	27.08 (31.76)	H = 34.491 **P < 0.001**
	**Outdoor**	12.50 (20.83)	
	**Mixed**	16.17 (22.40)	
	**Non-Smoker**	10.42 (18.75)	

* Values in bold indicate significant difference (P < 0.05).

Table 3: Kruskal-Wallis test results for the usage of the eye cosmetics products.

**Variable**		**Median OSDI (IQR)**	**Kruskal-Wallis Test***
**Inner Eyeliner**	**Daily**	19.79 (27.09)	H = 17.329
	**3-4 times per week**	14.58 (15.62)	**P = 0.002**
	**1-2 times per week**	16.67 (28.64)	
	**1-2 times per month**	16.67 (21.87)	
	**Never/NA**	12.50 (18.75)	
**External Eyeliner**	**Daily**	16.67 (26.04)	H = 23.263
	**3-4 times per week**	16.67 (28.12)	**P < 0.001**
	**1-2 times per week**	15.63 (29.17)	
	**1-2 times per month**	12.50 (20.83)	
	**Never/NA**	10.42 (16.66)	
**False Eyelashes**	**Daily**	14.58 (48.96)	H = 22.444
	**3-4 times per week**	33.33 (40.63)	**P < 0.001**
	**1-2 times per week**	20.83 (29.17)	
	**1-2 times per month**	21.87 (28.13)	
	**Never/NA**	12.50 (16.67)	
**Eye shadow**	**Daily**	17.71 (24.48)	H = 17.001
	**3-4 times per week**	12.50 (22.92)	**P = 0.002**
	**1-2 times per week**	16.67 (26.04)	
	**1-2 times per month**	14.58 (20.32)	
	**Never/NA**	10.42 (17.18)	

**Mascara**	**Daily**	14.58 (22.92)	H = 20.234<br><br>**P < 0.001**
	**3-4 times per week**	14.58 (18.75)	
	**1-2 times per week**	16.67 (31.25)	
	**1-2 times per month**	18.75 (37.50)	
	**Never/NA**	10.42 (16.66)	

* Values in bold indicate significant difference ($P < 0.05$).

Table 4: Kruskal-Wallis test results for eye cosmetics routines and practices.

**Variable**		**Median OSDI (IQR)**	**Kruskal-Wallis Test***
**Duration of Cosmetics use (per year)**	**< 6 months**	16.67 (25.00)	H = 22.628
	**6-12 months**	18.75 (9.17)	**P < 0.001**
	**>12 months**	14.58 (20.83)	
	**Don’t use or NA**	10.42 (16.66)	
**Duration of Cosmetics use (per day)**	**< 6 hours**	16.67 (20.32)	H = 18.821
	**6-12 hours**	14.58 (33.33)	**P < 0.001**
	**>12 hours**	14.58 (20.83)	
	**Don’t use or NA**	10.42 (16.66)	
**Cleansing Technique**	**Cleansing Cream**	14.58 (22.92)	H = 22.990
	**Soap Water**	12.50 (18.75)	**P < 0.001**
	**Water ONLY**	17.71 (34.90)	
	**Don’t use or NA**	10.42 (16.66)	
**Cleansing Rate (per day)**	**Rarely**	14.58 (20.83)	H = 35.622
	**Sometimes**	14.58 (31.25)	**P < 0.001**
	**Always**	33.33 (27.08)	
	**Don’t use or NA**	10.42 (16.66)	
	**Sleep with lenses**	28.13 (33.85)	H = 6.937
	**Sleep with eye cosmetics**	23.96 (40.62)	

**Sleep Routine**	**Sleep with eye cosmetics and lenses**	52.08 (67.71)	P = 0.074
	**Sleep with no lenses and eye cosmetics or NA**	12.50 (18.75)	

*Values in bold indicate significant difference (P < 0.05).

Table 5: Spearman's rank correlation results.

**Dependent Variable**	**Independent Variables**	**Correlation Coefficient**	**P-value***
OSDI Score	Electronic device usage per day (in hours)	0.694	**<0.001**
	Studying hours per day	0.661	**<0.001**
	Number of smoking years	0.380	**<0.001**
	Number of cigarettes smoked per week	0.175	**<0.001**
	Number of Dokha smoked per week	0.005	0.896
	Number of Shisha smoked per week	0.097	**0.013**

*Values in bold indicate significant difference ($P < 0.05$).

Click here to access/download
Supporting Information
Supporting information.docx
