# Supplementary material for: Behavioral and cultural determinants of symptomatic dry eye disease among university students in the UAE
Source: PLoS One. 2026 Feb 26;21(2):e0343508. doi: 10.1371/journal.pone.0343508 (PMC12944768; doi:10.1371/journal.pone.0343508)
Supplement: Attachment — Submitted filename: Response to Reviewers.docx [file pone.0343508.s005.docx]

Reviewer #2: I would like to thank the authors for revising the manuscript. In the revision, the authors successfully highlighted the importance of this study. However, there are some remaining points that may further improve the quality of the manuscript.

1. When considering the inclusion criteria and the response letter, 654 participants do not refer to the total population. The target population should be all students aged 18 years or older. The authors should revise the sentence in line 113. Revised

2. Add the “English version of OSDI questionnaire” in line 124. Revised

3. Line 132 – 133, and 236-238 should be revised and moved to data analysis section. Revised

4. Line 157 – 158 should be moved to section 2.2 Revised.

5. The presentation of results lacks consistency; some sections report mean ranks, whereas others report median OSDI scores. Consistency throughout the section is recommended.

We respectfully note that the variation in reporting statistics reflects the different statistical tests applied. For non-parametric tests (Kruskal-Wallis), we report median and interquartile ranges as SPSS outputs these as the appropriate measures for non-normally distributed data. For parametric comparisons, we report means as recommended by a reviewer in the previous revision round.

6. There are 3.2 two times. Revised

7. The authors should consider restructuring the presentation of the results. For instance, the findings on smoking are fragmented between Tables 2 and 5. Grouping these related points together would improve the narrative flow. Additionally, this would prevent the smoking-related data from incorrectly appearing under the 'Eye Cosmetics Routines and Practices' section Revised

8. To clarify the data, the table 3 and 4 should add N(%) into each variable. @Mona

We have revised now Tables 3 and 4 and included frequency counts and percentages [N (%)] for each categorical variable, consistent with the format used in Table 1. This will improve transparency by showing the distribution of participants across each behavioral and cosmetic exposure category.

9. It is unclear if categorizing OSDI scores into 'dry eye' and 'normal' groups for OR and CI analysis is more effective for meeting the study objectives than analyzing the non-normally distributed OSDI scores directly. Could the authors please justify this approach?

We dichotomized OSDI scores for two key reasons:

First, our primary objective was to identify risk factors for the *presence* of dry eye disease rather than to examine symptom severity. Dichotomization using the established OSDI cutoff of ≥[insert your cutoff: 13 or 23] directly addresses this diagnostic question and aligns with clinical decision-making.

Second, this approach provides clinically interpretable results. The odds ratios from logistic regression offer practitioners straightforward estimates of how risk factors affect the likelihood of having dry eye disease—the same framework clinicians use when applying OSDI thresholds in practice.

While we acknowledge some loss of statistical information with dichotomization, we believe this is justified given our study aims and the clinical utility of categorical results for translation into practice.

**Reviewer #4: Reviewer’s comments**

I have added the comments in each previous response with additional comments as follows:

Reviewer #4: First of all, your research had very detailed information and I enjoyed reading your manuscript. Thank you for describing different lifestyle and cultures in the Middle East for the reader to have better understanding of your research purposes and outcomes. However, I have the following comments and questions:

No 1. Regarding the cosmetic use, you have analysed each type of cosmetic as an isolated factor. However, I believe that many individuals have worn more than one type of cosmetic on their eyes, including eyeliner and mascara. Do you think there is an alternative analysis for those associated/multivariable factors, rather than the analysis of each factor separately? Also, the preference for cosmetic use in females, but not males, may encourage the subgroup analysis of female participants in this issue.

Answer: While we acknowledge that many individuals use multiple eye cosmetic products simultaneously, we believe analyzing each cosmetic type separately is appropriate and methodologically justified for this exploratory study for several reasons.
First, examining each product individually allows identification of product-specific associations with OSDI scores, which is clinically relevant as different cosmetics vary in composition, application site, and potential ocular surface impact. Second, simultaneous inclusion of multiple cosmetic variables in a single model would likely introduce multicollinearity given the high co-occurrence of cosmetic practices, potentially compromising parameter stability and interpretability in our non-parametric analyses. Third, this approach is consistent with published epidemiological literature on eye cosmetics and dry eye symptoms, facilitating comparison with existing studies.
Additionally, our gender-stratified analyses confirmed that the observed associations were primarily driven by female participants, where cosmetic use was prevalent, strengthening the validity of our findings. While cumulative cosmetic exposure represents an important area for future research, the current approach is appropriate for this study's exploratory aims and provides clear, interpretable insights into individual cosmetic practices and dry eye symptoms.
COMMENTS: Thank you for your explanation. In terms of simplicity and comparability between studies, I agree with your reasons to choose each cosmetic type separately.
However, for the female predilection, as your manuscript title is “Behavioral and Cultural Determinants of Symptomatic Dry Eye Disease Among University Students in the UAE”, so your manuscript should provide the broad idea of overall UAE students.

Answer: We strongly agree with the reviewer regarding the focus of the manuscript on the overall UAE students. The authors have focused on the overall UAE students when investigating the prevalence of DED and its association with smoking. However, due to the gender-based use of eye cosmetics, the statistical analyses were not suitable for the overall UAE students (both genders).

No 2. Due to the design of the questionnaire survey, please also describe the response rate of the questionnaire in each field or describe how the questionnaire is designed to fill all questions as a mandatory rule to submit. Additionally, please also discuss the pros and cons of using a questionnaire.
Answer: As the data collection occurred via an online questionnaire, it was no possible to measure the response rate in each field. The google survey has reached hundreds of university students in the UAE (several field of studies and universities) while only the interested students responded via submitting their responses online. The google form is designed to fill all questions as a mandatory role to submit their responses. Pros include a wider reach out of university in all over the UAE. Cons include response bias or social desirability effects that may potentially influence the study findings; subjective findings that may not align with clinical sign; and selective recruitment of participants that may affect the generalizability of the study findings.
COMMENTS: I appreciated your study design and your capability to access to many university students around the UAE. At the beginning, I am just curious about the mandatory questions in the questionnaire, but you have already answered that all questions must be answered to submit. So, I am satisfied with this response. Thank you for your valuable comment.

No 3. Moreover, you have described/quantified many habits as longer, more frequent, shorter. Could you please describe them more in quantifiable measures? Also does weekly and monthly smokers mean once a week and once a month, respectively?
Answer: Authors have described all the recommended terms into a quantifiable measure in the tables and their relevant text. For example, the term “weekly” has been described by 1-2 times per week, and the term “monthly” has been described by 1-2 times/month.
COMMENTS: Thank you for make it clearer. I am satisfied with this response. Thank you for your valuable comment.

No 4. In lines 301 to 303, you emphasize the findings of “Dokha and Mouassal waterpipe users exhibited the highest median OSDI scores” and continuously describe the previous findings on smoking and oxidative stress. Does this specific type of smoking cause more oxidative stress than others?
Answer: Both Dokha and Mouassal (waterpipe/shisha) are associated with high oxidative stress, and in some contexts, they may produce equal or greater oxidative stress than cigarettes, though the mechanisms and exposure patterns differ. Dokha is often more intense per session than cigarette smoking due to its very high nicotine content (strong stimulation of oxidative pathways); rapid and deep inhalation (sharp spikes in reactive oxygen species; and its positive correlation with an oxidative stress marker (e.g., lipid peroxidation) (ref: Samara, F., Alam, I. A., & ElSayed, Y. (2022). Midwakh: Assessment of levels of carcinogenic polycyclic aromatic hydrocarbons and nicotine in dokha tobacco smoke. Journal of analytical toxicology, 46(3), 295-302). Additionally, Mouassal smoking causes prolonged and cumulative oxidative stress along with high exposure to carbon monoxide, polycyclic aromatic hydrocar (PAHs), and heavy metals that would generate systemic oxidative stress and inflammation (ref: Charab, M. A., Abouzeinab, N. S., & Moustafa, M. E. (2016). The protective effect of selenium on oxidative stress induced by waterpipe (narghile) smoke in lungs and liver of mice. Biological trace element research, 174(2), 392-401).
COMMENTS: Thank you for clarifying more details regarding the Dokha and Mouassal. Could you please briefly add these explanations in the discussion part? Added as recommended in the discussion.

No 5. The cleansing methods also prompt a question to me when you describe “Frequent cleansing or always” as a subgroup. Is it a usual habit to leave the cosmetics uncleaned for longer than a day? This is quite interesting in terms of cultural differences, as you mentioned.
Answer: Poor cosmetic hygiene (e.g., not cleaning makeup brushes, sponges, or removing makeup daily) is a global issue, documented across many countries and regions. In fact, Arab cultures have a strong emphasis on personal cleanliness and grooming, which is reflected in both daily routines and religious/cultural norm. Accordingly, there is no evidence that leaving cosmetics uncleaned for more than a day is a “usual habit” among UAE students. However, cosmetic hygiene practices vary widely among individuals and that would explain why this subgroup is investigated in our study.
COMMENTS: I do apologize, but the purpose of my comments was only to clarify the results. The title of your manuscript has led me to consider all factors as a subset of cultural and behavioral determinants. I do agree with your explanation of cosmetic hygiene, and I am satisfied with your responses. Thank you for your valuable comment.

No 6. I believe that many readers are unfamiliar with the H-value, please could you explain more in the manuscript on how to interpret the findings, in association with P-value, so all readers can follow your manuscript with more understanding?
Answer: We agree that many readers may be unfamiliar with the H-value (Kruskal-Wallis H statistic) and its interpretation. We have now added a clear explanation of the Kruskal-Wallis H statistic and its interpretation in the …?
COMMENTS: I am satisfied with the response. Thank you for your valuable comment.

Additional comments
1. In the method part (line 147-148), “The internal consistency of the behavioral and cultural risk items was assessed using Cronbach’s alpha, yielding a value of 0.856. This indicates good internal reliability and supports the use of these as a coherent measure in subsequent analyses.” Please specify what questionnaire you have used. Has it already been tested for validity and reliability beforehands as I have seen only the “Cronbach’s alpha” score.

The questionnaire used to assess behavioral and cultural risk factors has been previously validated and is described in the Introduction section (line 75) with full details available in reference 5.

The questionnaire was specifically developed and validated for assessing dry eye disease risk factors in our population. In addition to the prior validation work reported in reference 5, we conducted Cronbach's alpha analysis in the current study (α = 0.856) to confirm that the internal consistency remained robust in our specific sample.
